# A Comparison of Visual Outcomes and Patient Satisfaction Between Photorefractive Keratectomy and Femtosecond Laser-Assisted In Situ Keratomileusis

**DOI:** 10.7759/cureus.1641

**Published:** 2017-09-01

**Authors:** Nauman Hashmani, Sharif Hashmani, Priyanka Ramesh, Hina Rajani, Junaid Ahmed, Jaish Kumar, Arun Kumar, Munira Jamali

**Affiliations:** 1 Ophthalmology, Hashmanis Hospital; 2 Student, Dow Medical College, Karachi, Pakistan; 3 Dow University of Health Sciences, Civil hospital karachi

**Keywords:** femto second laser, lasik, photorefractive keratectomy, refractive surgery, visual outcomes

## Abstract

Purpose

To compare visual outcomes and satisfaction among patients of photorefractive keratectomy (PRK; Wavelight EX 500, Alcon, Ft Worth, TX, USA) and femtosecond laser-assisted in situ keratomileusis (FAL; Wavelight FS 200 laser and Wavelight EX 500, Alcon, Ft Worth, TX, USA).

Methods

We performed a retrospective study of 409 eyes in 207 patients that underwent either PRK (n=90) or FAL (n=117) at the two centers of Hashmanis Hospital, Karachi, Pakistan. The included refractive outcomes were sphere diopters (D), cylinder D, and spherical equivalent D. Additionally, visual acuities were included. All of these were assessed preoperatively and at the one-month postoperative check-up. Patient satisfaction was gauged at the time of chart review by contacting the patient.

Results

When looking at the postoperative outcomes, we found all values to be statistically significant (p<0.001) with superior outcomes in the FAL cohort. Additionally, 90% and 15% of eyes achieved a postoperative uncorrected visual acuity (UCVA) of 20/20 in FAL and PRK, respectively. Furthermore, the efficacy indexes of the FAL and PRK arms were 1.00 and 0.82, respectively. The predictability of the procedures were 92.1% and 64.9%, respectively. Lastly, 93.3% of patients were satisfied with FAL and 95.7% with PRK.

Conclusion

Our study shows superior visual outcomes in patients undergoing FAL. However, we found a higher satisfaction rate in those that underwent PRK, perhaps due to the higher cost of FAL.

## Introduction

The past few decades have witnessed several advancements in the field of refractive surgery. One procedure, photorefractive keratectomy (PRK; Wavelight EX 500, Alcon, Ft Worth, TX, USA), involves the use of an excimer laser to ablate the corneal stroma after epithelial debridement [1]. The successor to PRK is laser-assisted in situ keratomileusis (LASIK) in which an epithelial flap is created to ablate the underlying stroma [2-3]. Although many believe LASIK is a safer operative procedure with less corneal damage [4], surgeons argue that PRK improves visual acuity and prevents future development of ectasia [5]. For both procedures, however, screening those who are unsuitable to undergo the treatment is vital [6-7].

The initial flap in LASIK was traditionally created by a microkeratome. With recent advances, however, it has become possible to create flaps using a femtosecond laser; this variant is known as femto LASIK (FAL; Wavelight FS 200 laser and Wavelight EX 500, Alcon, Ft Worth, TX, USA). FAL creates planar flaps that significantly decrease flap associated complications. Additionally, a smaller deviation in flap thickness is noted when compared to microkeratomes [8]. One study found that PRK patients had an improved uncorrected distance visual acuity (UDVA) when compared to those undergoing FAL [9]. It was further noted that with increased pupil diameter, spherical, and higher-order aberrations increased prominently in PRK patients. Thus, it was concluded that in those undergoing FAL, spherical and higher-order aberrations were lower, but visual acuity was better in those undergoing PRK [9].

Although studies have evaluated postoperative results of LASIK in Pakistan [10-11], none have compared FAL with PRK. Additionally, this is the first study in the region to evaluate visual outcomes of PRK as well. Furthermore, this is one of the first studies in the world to include a wide range of refractive errors when comparing the two modalities.

## Materials and methods

Patients

We conducted a retrospective analysis of 409 eyes (207 patients) that underwent FAL and PRK at the Hashmanis Hospital, Karachi, Pakistan. Of these, 229 (117 patients) underwent FAL and 180 (90 patients) underwent PRK. The study period lasted from January 2014 to October 2016. All variables were recorded both preoperatively and at the one-month postoperative checkup. Three surgeons performed PRK while one performed FAL. Ethical approval was provided by the Ethics Committee of Hashmanis Hospital and patient consent was sought before carrying out the procedure.

Inclusion and exclusion criteria

For both procedures, our inclusion criteria were: age greater than 18 years with a stable refraction, central corneal thickness (CCT) greater than 480 μm, a presumed residual stromal bed greater than 250 μm, and discontinuing contact lens use for more than a week.

The exclusion criteria included any active or residual ocular pathology, like glaucoma or retinal dystrophy, and dry eyes with a Schirmer’s two test value below 2 mm. Furthermore, those who were immunocompromised, pregnant, or nursing were excluded as well.

All patients underwent the routine ophthalmological examinations prior to surgery. These included: uncorrected visual acuity (UCVA), best corrected visual acuity (BCVA), cycloplegic and subjective refractive error, slit lamp examination, dilated retinal exam, ultrasonic pachymetry, keratometry, and corneal topography.

Attempted refraction

The attempted postoperative refraction was emmetropia in all patients that were either greater than -12.00 diopters (D) or less than 6.00 D. For those with a greater refractive error preoperatively, the target was the refractive error subtracted from the above values. However, both targets were dependent on the keratometry reading.

The efficacy index was defined as the ratio of the mean postoperative UCVA to the mean preoperative BCVA. Predictability was the percentage of eyes that achieved the target spherical equivalent within ± 1.00 D. 

Procedure and postoperative care

In the FAL arm, a 120 μm flap was created using a Wavelight FS 200 laser and stromal ablation was performed by the Wavelight EX 500 machine. A tissue separator was used to raise the initial flap and the eye was irrigated using a balanced salt solution after flap replacement at the end of the procedure.

During PRK, we used 18% alcohol for 18 seconds to achieve a 9 mm zone of epithelial debridement. Then, we completed a central ablation of 6.5 mm with an excimer laser using a Wavelight EX 500 machine. Subsequent to the procedure, we used a bandage soft contact lens on the cornea.

After both surgeries, the patient was advised to follow certain postoperative measures. These included: artificial tears four times a day for three weeks, moxifloxacin eye drops four times a day for 10 days, and combination drops with tobramycin and dexamethasone, four times a day for 10 days. For PRK, we started fluoromethalalone eye drops four times daily for 15 days after stopping the combination drops of dexamethasone and tobramycin.

Patient satisfaction

Patient satisfaction was tabulated by contacting the patient at the time of chart review. We recorded the response in the following categories: “extremely satisfied”, “very satisfied”, “satisfied”, and “not satisfied”.

Statistical analysis

The analysis was performed using the Statistical Package for the Social Sciences (SPSS) (IBM Corp., Armonk, NY), version 23 software. The graphs and tables were constructed using Microsoft Excel (Microsoft Corp., Redmond, WA) and SPSS. The Shapiro-Wilk test was used to test for normality and the Mann-Whitney U test was used to compare the refractive outcomes. A P-value less than 0.05 was considered to be statistically significant.

## Results

General characteristics

A total of 409 eyes of 207 patients (147 females and 60 males) were treated. Of these, 229 eyes (117 patients; 36 males and 81 females) underwent FAL and 180 (90 patients; 24 males and 66 females) underwent PRK. This is seen in Table 1.

**Table 1:** Preoperative Data abbreviations: Y=years; M=male; F=female; D=diopters; PRK=photorefractive keratectomy; LASIK=laser in situ keratomileusis

Table 1: Preoperative Data			
Variable	PRK (n=180)	Femto LASIK (n=229)	P-value
Age (Y)	25.0 ± 5.8 (18.0 to 45.0)	27.0 ± 7.3 (18.0 to 52.0)	
Gender (M/F)	48/132	72/157	
Sphere (D)	-5.0 ± 2.7 (-14.5 to 2.0)	-4.3 ± 2.3 (-9.8 to 6.8)	0.574
Cylinder (D)	-1.0 ± 0.8 (-5.0 to 0.25)	-1.0 ± 1.3 (-12.8 to 3.3)	0.955
Spherical Equivalent (D)	-5.4 ± 2.7 (-15.5 to 0.0)	-4.5 ± 2.5 (-13.4 to 8.0)	0.678

Efficacy and predictability

For the PRK cohort, the preoperative and postoperative spherical means were -5.0 ± 2.7 D and -0.3 ± 1.5 D, respectively. For the FAL group, they were -4.3 ± 2.3 D and 0.3 ± 0.7 D, respectively. 

The cylinder preoperative and postoperative means for the PRK arm were -1.0 ± 0.8 D and -0.8 ± 0.6 D, respectively. For FAL, the values were -1.0 ± 1.3 D and, -0.5 ± 0.6 D, respectively.

Finally, the PRK cohort’s spherical equivalent preoperative and postoperative means were -5.4 ± 2.7 D and -0.6 ± 1.6 D, respectively. Comparatively, the FAL group’s means were -4.5 ± 2.5 D and 0.0 ± 0.7 D, respectively. All data mentioned above are represented in Tables 1-2.

**Table 2:** Postoperative Data abbreviations: D=diopters; PRK=photorefractive keratectomy; LASIK=laser in situ keratomileusis

Table 2: Postoperative Data			
Variable	PRK (n=180)	Femto LASIK (n=229)	P-value
Sphere (D)	-0.3 ± 1.5 (-5.5 to 2.3)	0.3 ± 0.7 (-5.5 to 1.8)	<0.001
Cylinder (D)	-0.8 ± 0.6 (-3.5 to 0.0)	-0.5 ± 0.6 (-5.0 to 1.0)	<0.001
Spherical Equivalent (D)	-0.6 ± 1.6 (-5.9 to 1.6)	0.0 ± 0.7 (-6.0 to 1.6)	<0.001

The preoperative values of the procedures were statistically insignificant in all cases. The postoperative values, on the other hand, were statistically significant.

**Figure 1:**
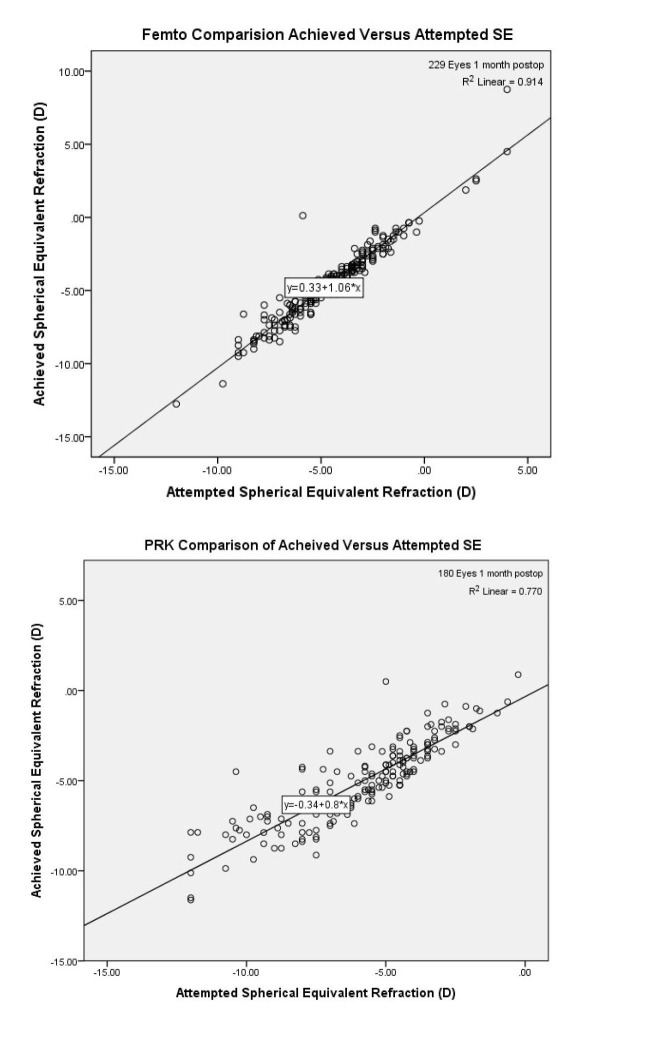
Comparison of attempted versus achieved spherical equivalent at one-month postoperative checkup abbreviations: PRK=photorefractive keratectomy; SE=spherical equivalent; D=diopters

**Figure 2:**
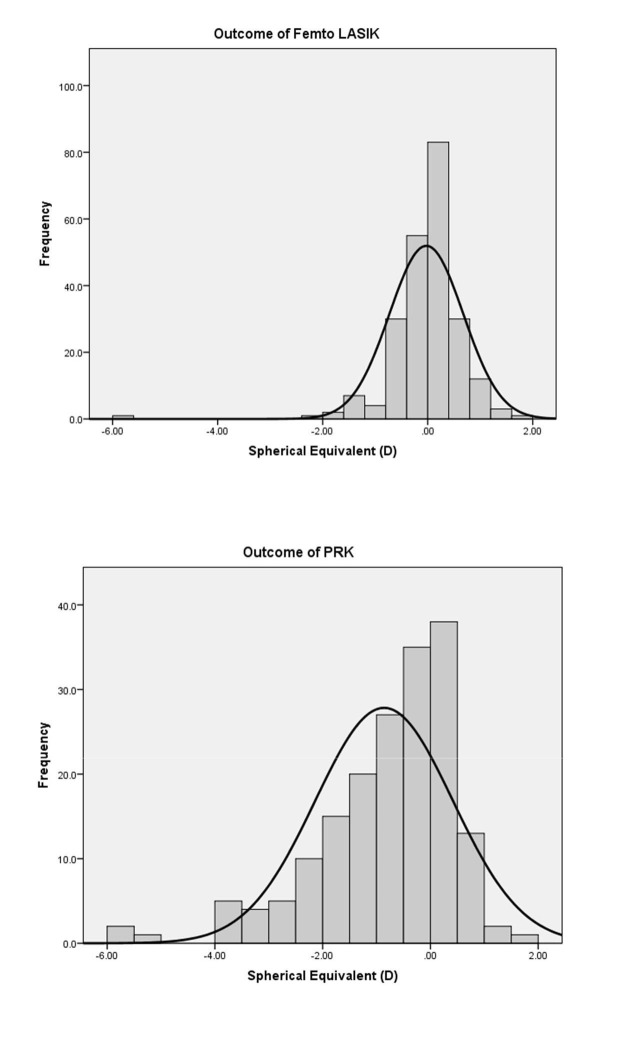
Frequency of refractive outcomes at one-month postoperative checkup abbreviations: PRK=photorefractive keratectomy; LASIK=laser in situ keratomileusis; SE=spherical equivalent; D=diopters

**Figure 3:**
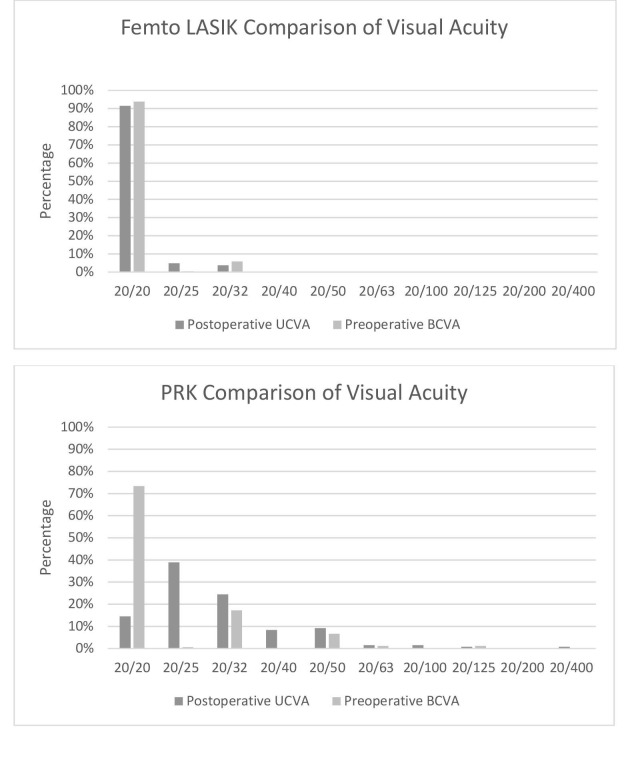
Comparison of postoperative UCVA to preoperative BCVA at one-month postoperative checkup abbreviations: PRK=photorefractive keratectomy; LASIK=laser in situ keratomileusis; UCVA=uncorrected visual acuity; BCVA=best corrected visual acuity

**Figure 4:**
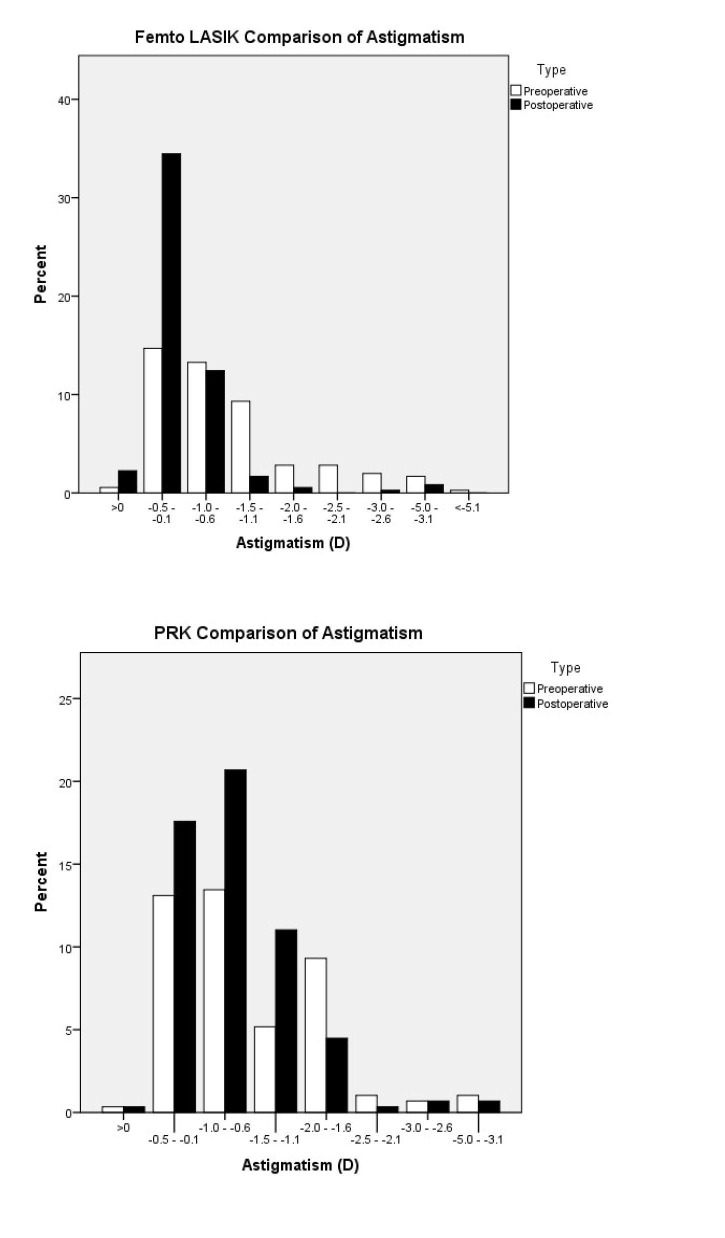
Comparison of astigmatic outcomes at one-month postoperative checkup abbreviations: PRK=photorefractive keratectomy; LASIK=laser in situ keratomileusis; D=diopters

As shown in Figure 1, 90% of eyes that underwent FAL obtained a postoperative UCVA of 20/20. On the other hand, only 15% of eyes in the PRK arm boasted a 20/20 postoperative UCVA. Additionally, predictability was 92.1% for FAL and 64.9% for PRK. Figure 2 shows the spherical equivalent outcomes of the two procedures. Furthermore, the efficacy index of PRK and FAL were 0.82 and 1.00, respectively. Figures 3-4 compare the achieved versus attempted spherical equivalent and astigmatism, respectively.

Patient satisfaction

Out of the 75 FAL patients, 93.3% were satisfied (n=70), of which, 13.3% (n=10) were extremely satisfied, as shown in Table 3. On the other hand, out of the 46 PRK patients, 95.7% reported satisfaction (n= 44) of which, 15.2% (n=7) were extremely satisfied. Additionally, 4.3% (n=2) of PRK patients and 6.7% (n=5) of FAL patients were not satisfied with the procedure.

**Table 3:** Patient Satisfaction *= number of patients abbreviations: PRK=photorefractive keratectomy; LASIK=laser in situ keratomileusis

Table 3: Patient Satisfaction				
	PRK (n=46*)		Femto LASIK (n=75*)	
Satisfaction Level	Total	Percentage	Total	Percentage
Extremely Satisfied	7	15.2	10	13.3
Very Satisfied	14	30.4	30	40.0
Satisfied	23	50.0	30	40.0
Not Satisfied	2	4.3	5	6.7

## Discussion

PRK has been compared with a variety of LASIK techniques; for example, LASIK performed with a microkeratome [12] or FAL [13]. However, no consensus has been established on the superior procedure between PRK and FAL. Although previous studies have demonstrated a quicker and less painful recovery in patients undergoing FAL, the final postoperative outcome has been found to be similar among the two modalities [12-13]. This study aimed to investigate the postoperative visual outcomes of the two procedures.

Sajjadi, et al. and AlMahmoud, et al. found no statistically significant difference in the refractive outcomes between the two procedures [9, 14], unlike our study. However, numerous other studies agree with our findings [13, 15-16]. Interestingly, both AlMahmoud, et al. and Sajjadi, et al. had narrow preoperative refractive ranges in their cohorts; they included only mild to moderate myopic patients. This was unlike our study which catered to a wider range of refractive errors, including high myopes. This could explain the difference in the outcomes as it has been seen in multiple studies that FAL is superior in those with high myopia [15, 17].

In this study, 90% of FAL patients achieved a UCVA of 20/20, whereas,­­­­­­­­­­ only 15% of PRK patients achieved this outcome. Several studies agree with our findings as well [13]. AlMahmoud, et al., for example, reported that 15 eyes (11%) attained a 20/12.5 visual acuity or better in the FAL cohort compared to four (3%) in the PRK group [14]. Not all studies agree with us: Sajjadi, et al. found no difference in the visual acuity between the two groups [9]. Another study, a randomized control trial, proposed that while FAL is better in the short-term, there is no difference in the visual acuity between the groups at six months [12]. We recommend further investigation into this matter as there are no consistent findings among the various studies.

In our population, the PRK and FAL efficacy indexes were maintained at 0.82 and 1.00, respectively. One meta-analysis conducted in 2006 concluded that the FAL arm had superior efficacy when compared to that of PRK [18]. However, this study included data that was published before 2001 and concluded that the findings did not reflect the results of modern technology. Another meta-analysis was then conducted in 2013. They concluded that while the recovery in patients was quicker in the FAL arm, there was no difference in the efficacy one-year post surgery [19]. This is further supported by a retrospective analysis performed in 2016 [13]. Our study had a limited follow-up and could not account for the efficacy at one-year and perhaps a longer study would yield similar results to previous research.

It was interesting to note that a greater percentage of patients were satisfied with PRK despite superior outcomes in the FAL arm. This can be explained by the significantly higher cost of FAL and, therefore, greater expectations from the procedure. We recommend a cost analysis of FAL to determine the efficiency of using this procedure at its current price.

There are several limitations to consider in this study. Firstly, this is a retrospective analysis and all associated limitations apply. Secondly, the prevalence of higher order aberrations could not be assessed due to technological deficiencies at our facility. Thirdly, this was a short-term study, and therefore, outcomes in the long-term could not be assessed. Lastly, we did not assess the complications of the two procedures.

## Conclusions

Our study shows superior visual outcomes in patients undergoing FAL. However, we found a higher satisfaction rate in those that underwent PRK, perhaps due to the higher cost of FAL.
